# Biomechanics and anterior cruciate ligament reconstruction

**DOI:** 10.1186/1749-799X-1-2

**Published:** 2006-09-25

**Authors:** Savio L-Y Woo, Changfu Wu, Ozgur Dede, Fabio Vercillo, Sabrina Noorani

**Affiliations:** 1 Musculoskeletal Research Center, Department of Bioengineering, University of Pittsburgh, Pennsylvania, USA

## Abstract

For years, bioengineers and orthopaedic surgeons have applied the principles of mechanics to gain valuable information about the complex function of the anterior cruciate ligament (ACL). The results of these investigations have provided scientific data for surgeons to improve methods of ACL reconstruction and postoperative rehabilitation. This review paper will present specific examples of how the field of biomechanics has impacted the evolution of ACL research. The anatomy and biomechanics of the ACL as well as the discovery of new tools in ACL-related biomechanical study are first introduced. Some important factors affecting the surgical outcome of ACL reconstruction, including graft selection, tunnel placement, initial graft tension, graft fixation, graft tunnel motion and healing, are then discussed. The scientific basis for the new surgical procedure, i.e., anatomic double bundle ACL reconstruction, designed to regain rotatory stability of the knee, is presented. To conclude, the future role of biomechanics in gaining valuable in-vivo data that can further advance the understanding of the ACL and ACL graft function in order to improve the patient outcome following ACL reconstruction is suggested.

## Background

An anterior cruciate ligament (ACL) rupture is one of the most common knee injuries in sports. It is estimated that the annual incidence is about 1 in 3,000 within the general population in the United States, which translates into more than 150,000 new ACL tears every year [1,2]. Unlike many tendons and ligaments, a mid-substance ACL tear cannot heal and the manifestation is moderate to severe disability with "giving way" episodes in activities of daily living, especially during sporting activities with demanding cutting and pivoting maneuvers. Further, it can cause injuries to other soft tissues in and around the knee, particularly the menisci, and lead to early onset osteoarthritis of the knee. Therefore, surgical treatment using tissue autografts or allografts is frequently performed by surgeons on patients with a ruptured ACL. It is estimated that approximately 100,000 primary ACL reconstruction surgeries are performed annually in the United States [1,3]. The direct cost for these operations is estimated to be over $2 billion [4].

The goal of an ACL reconstruction is to reproduce the functions of the native ACL. Over the past three decades, clinically relevant biomechanical studies have provided us with important data on the ACL, particularly on its complex anatomy and functions in stabilizing the knee joint in multiple degrees of freedom (DOF). As such, surgical reconstruction of the ACL has not been able to reproduce its complex function. Both short and long term clinical outcome studies reveal an 11–32% less than satisfactory outcome for patients [5-8], among whom up to 10% may require revision ACL reconstruction [9]. Indeed, ACL reconstruction remains a significant clinical problem to date as there have been over 3,000 papers published in the last 10 years, with over half focusing on techniques, a large number on complications and related issues, and only a small percentage on clinical outcome.

This review paper will provide a perspective on how biomechanics has helped in understanding the complex function of the normal ACL as well as in advancing ACL reconstruction. Firstly, the anatomy and function of the ACL as well as available tools in ACL-related biomechanical study are briefly introduced. Secondly, the contributions of biomechanics in determining some key factors that affect the surgical outcomes of ACL reconstruction are discussed. Thirdly, the role of biomechanics in developing a new ACL reconstruction procedure, i.e., anatomic double bundle ACL reconstruction, is presented. Finally, the future role of biomechanics in gaining the needed in-vivo data to further improve the results of ACL reconstruction for better patient outcome is suggested.

## Anatomy and biomechanics of the ACL

The ACL extends from the lateral femoral condyle within the intercondylar notch, to its insertion at the anterior part of the central tibial plateau. The cross-sectional areas of the ACL at the two insertion sites are larger than those at the mid substance. The cross-sectional shape of the ACL is also irregular[10]. Functionally, the ACL consists of the anteromedial (AM) bundle and the posterolateral (PL) bundle [11]. It has been shown that the AM bundle lengthens and tightens in flexion, while the PL bundle does the same in extension [12]. These complex anatomies make the ACL particularly well suited for limiting excessive anterior tibial translation as well as axial tibial and valgus knee rotations.

Laboratory studies have determined load-elongation curve of a bone-ligament-bone complex by a uniaxial tensile test. The stiffness and ultimate load are obtained to represent its structural properties. In the same test, a stress-strain relationship can also be obtained, from which the modulus, tensile strength, ultimate strain, and strain energy density can be measured to represent the mechanical properties [13]. In addition, forces in the ACL can be measured by studying the knee kinematics in 6 DOF in response to externally applied loads. For instance, when a knee is subjected to an anterior tibial load, it undergoes anterior tibial translation, as well as internal tibial rotation. Thus, biomechanics is useful to determine the inter-relationships between the ACL and knee kinematics as the data serve as the basis for the goal of a replacement graft.

## Discovery of tools for biomechanical studies of the ACL and ACL grafts

There have been many tools, including buckle transducers, load cells, strain gauges, and so on, designed to measure the forces within the ACL when a load is applied to the knee [14-19]. All have contributed significantly to the knowledge of the function of the ACL. However, they all make contact with the ACL.

Other investigators prefer to measure the force in the ACL without contact. These include the use of radiographic or kinematic linkage systems attached to the bones and determine the forces in the ACL by combining kinematic data from the intact knee and the load-deformation curves of the ACL [12,20]. More recently, computer modeling and simulations have also been used to estimate the forces in the ACL during gait [21].

**Figure 1:**
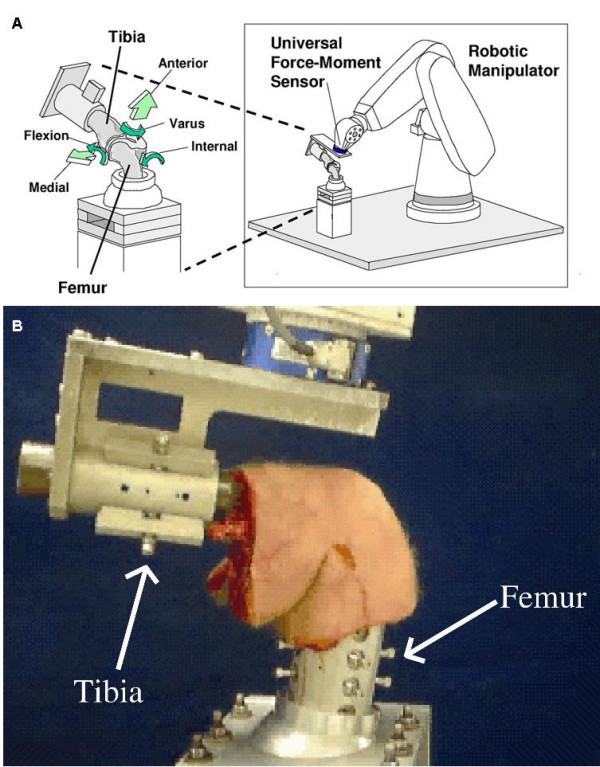
(a) The robotic/universal force-moment sensor (UFS) testing system designed to measure knee kinematics and in situ forces in 6 DOF. (b) A human cadaveric knee specimen mounted on the robotic/UFS testing system.

In our research center, we have pioneered the use of a robotic manipulator together with a 6-DOF universal force-moment sensor (UFS), as illustrated in Figure 1[22]. This robotic/UFS testing system can be used to measure the *in situ *force vectors of the ACL and the ACL graft in response to applied loads to the knee. This system is capable of accurately recording and repeating translations and rotation of less than 0.2 mm and 0.2°, respectively [23]. Interested readers may refer to Woo, et al. for the principles and detailed operation of this testing system [22,24].

**Figure 2:**
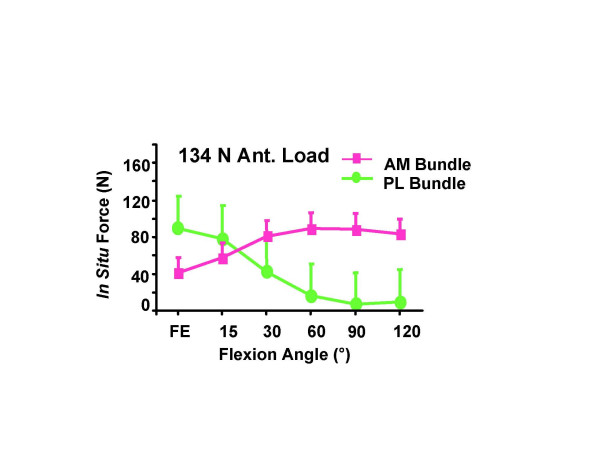
Magnitude of the in situ force in the intact AM bundle and PL bundle in response to 134 N anterior tibial load (mean ± SD and n = 10). (Reproduced with permission from Gabriel MT, Wong EK, Woo SL, Yagi M, Debski RE: Distribution of in situ forces in the anterior cruciate ligament in response to rotatory loads. J Orthop Res 2004, 22:85–89).

Through the use of the robotic/UFS testing system, a thorough understanding of the function of the ACL, and more importantly its AM and PL bundles, was possible. For instance, it has been found that under an anterior tibial load, the PL bundle actually carried a higher load than the AM bundle with the knee near extension, and the AM bundle carried a higher load with the knee flexion angle larger than 30° (Figure 2) [25]. It was also found that when the knee was under combined rotatory loads of valgus and internal tibial torques, the AM and PL bundles almost evenly shared the load at 15° of knee flexion [25]. Thus, it is clear that the smaller PL bundle does play a significant role in controlling rotatory stability due to its more lateral femoral position.

## ACL reconstruction

The first intra-articular ACL reconstruction began with Hey-Groves in 1917; however, it was made popular by O'Donoghue in 1950. The introduction of arthroscopic equipment has further led to revolutionary changes in ACL surgery [26-28]. There has since been a significant increase in the frequency of ACL reconstruction as well as research on this procedure.

### Biomechanics for ACL reconstruction

**Figure 3:**
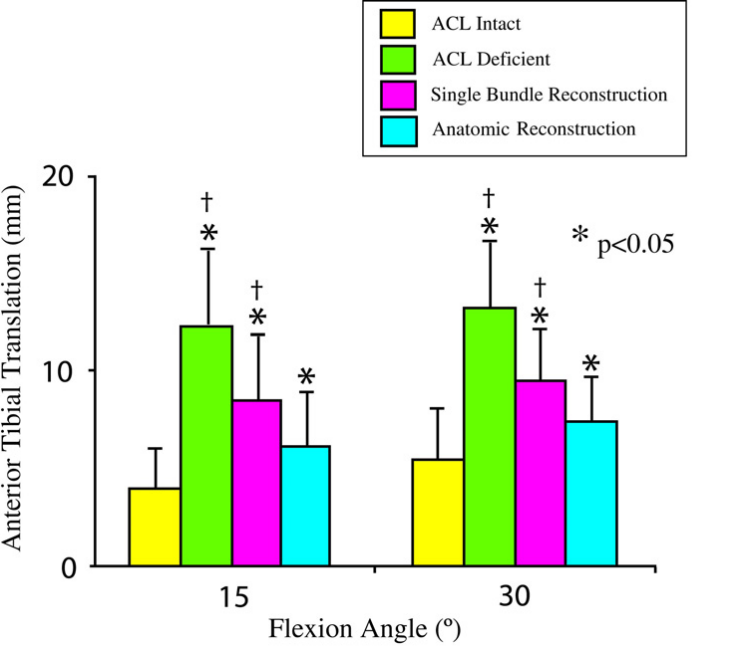
Coupled anterior tibial translation in response to combined 5-Nm internal tibial torque and 10-Nm valgus torque for 1) the intact, 2) ACL-deficient, and 3) ACL-reconstructed knee. * indicates significant difference when compared with the intact knee, † indicates significant difference when compared with the anatomic reconstruction (mean ± SD and n = 10). (Reproduced with permission from Yagi M, Wong EK, Kanamori A, Debski RE, Fu FH, Woo SL: Biomechanical analysis of an anatomic anterior cruciate ligament reconstruction. Am J Sports Med 2002, 30:660–666.)

**Figure 4:**
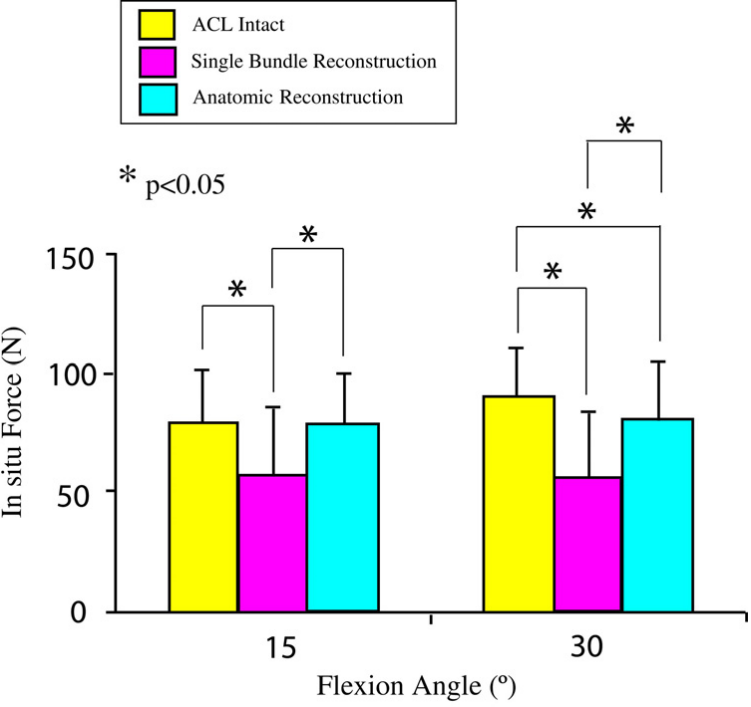
In situ force in the ACL and the replacement grafts in response to a combined rotatory load of 5-Nm internal tibial torque and 10-Nm valgus torque at 15° and 30° knee flexions (mean ± SD and n = 10). (Reproduced with permission from Yagi M, Wong EK, Kanamori A, Debski RE, Fu FH, Woo SL: Biomechanical analysis of an anatomic anterior cruciate ligament reconstruction. *Am J Sports Med *2002, 30:660–666.)

The ultimate aim of an ACL reconstruction is to restore the function of the intact ACL. Laboratory study on human cadaveric knee designed to evaluate the effectiveness of ACL reconstruction under clinical maneuvers, i.e anterior drawer and Lachman test, reveal that most of the current reconstruction procedures are satisfactory during anterior tibial loads [29]. However, they fail to restore both the kinematics and the *in situ *forces in the ACL under rotatory loads (Figures 3 and 4) and muscle loads [30,31].

### Factors affecting the outcome of an ACL reconstruction

**Figure 5:**
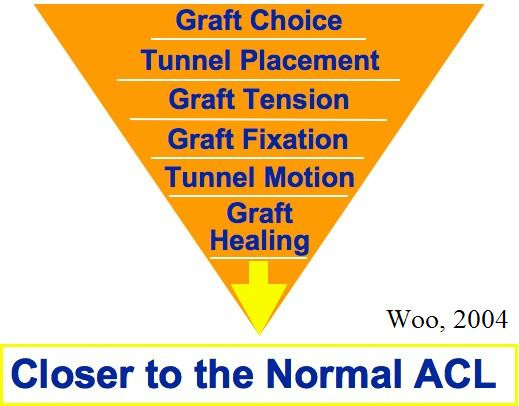
A logical sequence of factors to be considered in ACL reconstruction in order to improve the results.

Factors that could determine the fate of an ACL reconstruction include graft selection, tunnel placement, initial graft tension, graft fixation, graft tunnel motion, and rate of graft healing. We believe that there is a logical sequence to examine these factors in order to achieve the ideal results (Figure 5).

#### Graft selection

Over the years, a variety of autografts and allografts have been used for ACL reconstruction. Synthetic grafts had also been tried and are seldom used because of poor results. For autografts, the bone-patellar tendon-bone (BPTB) and hamstrings tendons are the most common, albeit some surgeons also use the quadriceps tendon and the iliotibial band. BPTB autografts have been proclaimed as the "gold standard" in ACL reconstruction. Recently, issues relating to donor site morbidity, such as arthrofibrosis, kneeling/patello-femoral pain, and quadriceps weakness, have caused a paradigm shift from 86.9% to 21.2% between 2000 to 2004 to quadrupled semitendinosus and gracilis tendon (QSTG) autografts [32,33].

Biomechanically, a 10-mm wide BPTB graft has stiffness and ultimate load values of 210 ± 65 N/mm and 1784 ± 580 N, respectively [34], which compare well with those of the young human femur-ACL-tibia complex (FATC) (242 ± 28 N/mm and 2160 ± 157 N, respectively) [35]. It also has the advantage of having bone blocks available for graft fixation in the osseous tunnels that leads to better knee stability for earlier return to sports. The QSTG autograft, evolved from a single-strand semitendinosus tendon graft, has very high stiffness and ultimate load values of (776 ± 204 N/mm, 4090 ± 295 N, respectively) [36]. Issues relating to graft tunnel motion and a slower rate of tendon to bone healing, as well as the reduction of hamstring function (to reduce anterior tibial translation) are of concern [37,38].

#### Tunnel placement

Femoral tunnel placement will have a profound effect on knee kinematics. In recent years, most surgeons choose to move the femoral tunnel to the footprint of the AM bundle of the ACL, i.e., near the 11 o'clock position on the frontal view of a right knee. Biomechanical studies have suggested that this femoral tunnel placement could not satisfactorily achieve the needed rotatory knee stability, whereas a more lateral placement towards the footprint of the PL bundle, i.e., the 10 o'clock position yielded better results [39]. Further, in addition to the frontal plane (i.e., the clock position), the tunnel position in the sagittal plane must also be considered [40]. In revision ACL surgery, it was discovered that there were a large percentage of wrong graft tunnel placement in this plane [41]. Still, it has been shown that there is no single position that could produce the rotatory knee stability close to that of the intact knee [39]. As a result, biomechanical studies have been conducted to evaluate an anatomic double bundle ACL reconstruction. The details will be discussed in a later section.

#### Initial graft tension

Laboratory studies have found that an initial graft tension of 88 N resulted in an overly constrained knee; while a lower initial graft tension of 44 N would be more suitable [42]. On the contrary, an *in vivo *study on goats found no significant differences in knee kinematics and *in situ *forces, between high (35 N) and low (5 N) initial tension groups at 6 weeks after surgery [43]. Viscoelastic studies revealed that the tension in the graft can decrease by as much as 50% within a short time after fixation because of its stress relaxation behavior [44,45]. More recently, a 2-year follow up study evaluating a range of graft tensions of 20 N, 40 N, and 80 N found that the highest graft tension of 80 N produced a significantly more stable knee (p < 0.05) [46]. Thus, the literature is confusing and definitive answers on initial graft tension remain unknown [47].

#### Graft fixation

There are advocates of early and aggressive postoperative rehabilitation as well as neuromuscular training to help athletes return to sports as early as possible [26]. To meet these requirements, increased rigidity of mechanical fixation of the grafts has been promoted and a wide variety of fixation devices are now available.

Biomechanically speaking, for a tendon graft with a bone block on one or both ends (e.g., quadriceps tendon, Achilles tendon, and BPTB), interference screws have been successfully used [48,49]. An interference screw fixation has an initial stiffness of 51 ± 17 N/mm [50], only about 25% of that of the intact ACL. Such fixation can be at the native ligament footprint (at the articular surface) and thus can limit graft-tunnel motion and increase knee stability. New interference screws with blunt threads have also been used for soft tissue grafts in the bony tunnel with minimal graft laceration. Recently, bioabsorbable screws have become available. They have stiffness and ultimate load values of 60 ± 11 N/mm and 830 ± 168 N, respectively, which are comparable to those for metal screw fixation [51-54]. The advantages of these screws are that they do not need to be removed in cases of revision or arthroplasty, or for MRI. The disadvantages include possible screw breakage during the insertion, inflammatory response, and inadequate fixation due to early degradation of the implant before graft incorporation in the bone tunnel [55-57].

Another type of fixation is the so-called "suspensory fixation", such as the use of EndoButton^® ^(Smith & Nephew, Inc., Andover, MA) to fix the graft at the lateral femoral cortex. The reported stiffness and ultimate load were 61 ± 11 N/mm and 572 ± 105 N, respectively [58]. Cross-pin fixation, such as TransFix^® ^(Arthrex, Inc., Naples, FL), is another method, and has a stiffness and ultimate load of 240 ± 74 N/mm and 934 ± 296 N, respectively [59]. It should be noted that as the graft is fixed further from the joint surface, the graft tunnel motion will increase. For the tibial side, cortical screws and washers are used. The ultimate load of the fixation is around 800–900 N [60,61].

In addition to the devices, the selection of knee flexion angle for graft fixation is also an important biomechanical consideration. It has been shown fixing the graft at full knee extension helps with the range of knee motion, while fixing at 30° of knee flexion increases the knee stability [62].

#### Tunnel motion

A goat model study showed that a soft tissue graft secured by an EndoButton^® ^and polyester tape can yield up to 0.8 ± 0.4 mm longitudinal graft tunnel motion and 0.5 ± 0.2 mm transverse motion [38]. In contrast, using a biodegradable interference screw could reduce these motions to 0.2 ± 0.1 mm and 0.1 ± 0.1 mm, respectively. In addition, the anterior tibial translation in response to an anterior tibial load for the EndoButton^® ^fixation was significantly larger than those fixed with a biointerference screw (5.3 ± 1.2 mm and 4.2 ± 0.9 mm, respectively. p < 0.05) [38]. Our research center has further demonstrated that with EndoButton^® ^and polyester tape fixation, the elongation of the hamstring graft under cyclic tensile load (50 N), was between 14–50% of the total graft tunnel motion, suggesting that the majority of motion came from the tape [63].

#### Graft-tunnel healing

Early and improved graft-tunnel healing is obviously desirable. Grafts that allow for bone-to-bone healing generally heal faster, i.e., 6 weeks. In contrast, soft tissue grafts require tendon-to-bone healing and take 10–12 weeks [64,65]. Animal model studies showed that the stiffness and ultimate load of the bone patellar tendon-bone autograft healing in rabbits at 8 weeks were 84 ± 18 N/mm and 142 ± 34 N, respectively, which were significantly higher compared to 45 ± 9 N/mm and 99 ± 26 N, respectively, for the tendon autograft healing (p < 0.05) [66].

Various biologically active substances have been used to accelerate graft healing. Bone morphogenetic protein-2 was delivered to the bone-tendon interface using adenoviral gene transfer techniques (AdBMP-2) in rabbits. The results showed that at 8 weeks, the stiffness and ultimate load (29 ± 7 N/mm and 109 ± 51 N, respectively) increased significantly, as compared to only 17 ± 8 N/mm and 45 ± 18 N, respectively, for untreated controls (p < 0.05) [67]. Exogenous transforming growth factor-β and epidermal growth factor have also been applied in dog stifle joints to enhance BPTB autograft healing after ACL reconstruction. At 12 weeks, the stiffness and ultimate load of the femur-graft-tibia complex reached 94 ± 20 N/mm and 303 ± 108 N, respectively, almost doubling those of the control group (54 ± 18 N/mm and 176 ± 74 N, respectively) [68]. Recently, periosteum has been sutured onto the tendon and inserted into the bone tunnel, resulting in superior and stronger healing [69]. These positive results have led to more studies on specific growth factors, time of application, and dosage levels so that clinical application can be a reality.

## A developing trend for ACL reconstruction

As traditional single bundle ACL reconstruction could not fully restore rotatory knee stability, investigators have explored anatomic double bundle ACL reconstruction for ACL replacement [70-73]. An anatomic double bundle ACL reconstruction utilizes two separate grafts to replace the AM and PL bundles of the ACL. Biomechanical studies have revealed that an anatomic double bundle ACL reconstruction has clear advantages in terms of achieving kinematics at the level of the intact knee with concomitant improvement of the *in situ *forces in the ACL graft closer to those of the intact ACL, even when the knee is subjected to rotatory loads [30]. Shown in Figures 3 and 4 are the coupled anterior tibial translation and the in situ force in the ACL and ACL grafts in response to combined rotatory loads of 5 N-m internal tibial torque and 10 N-m valgus torque. It is worth noting that the coupled anterior tibial translation after anatomic double bundle ACL reconstruction was 24% less than that after traditional single bundle ACL reconstruction. In addition, the in situ force in the ACL graft was 93% of the intact ACL as compared to only 68% for single bundle ACL reconstruction.

Of course, anatomic double bundle ACL reconstruction involves more surgical variables which could affect the final outcome. One of the major concerns is the force distribution between the AM and PL grafts and the potential of overloading either one of the two grafts [25]. Shorter in length and smaller in diameter, the PL graft would have a higher risk of graft failure. To find a range of knee flexion angles for graft fixation that would be safe for both of the grafts, our research center has performed a series of experiments and has discovered that when both the AM and PL grafts were fixed at 30°, the *in situ *force in the PL graft was 34% and 67% higher than that in the intact PL bundle in response to an anterior tibial load and combined rotatory loads, respectively. Meanwhile, when the AM graft was fixed at 60° and the PL graft was fixed at full extension, the force in the AM graft was 46% higher than that in the intact AM bundle under an anterior tibial load [74]. A follow-up study found that when the PL graft was fixed at 15° and the AM graft was fixed at either 45° or 15° of knee flexion, the *in situ *forces in the AM and PL grafts were below those of the AM and PL bundles, i.e., neither graft was overloaded. Thus, these flexion angles are safe for graft fixation [75].

## Future roles of biomechanics in ACL reconstruction

**Figure 6:**
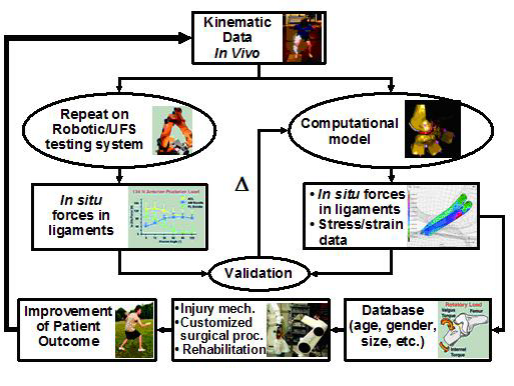
A flow chart detailing a combined approach of experiment and computational modeling based on *in vivo *kinematics. (Reproduced with permission from Woo SL, Debski RE, Wong EK, Yagi M, Tarinelli D: Use of robotic technology for diathrodial joint research. *J Sci Med Sport *1999, 2:283–297.)

In this review paper, we have summarized how *in vitro *biomechanical studies have made many significant contributions to the understanding of the ACL and ACL replacement grafts and how these data have helped the surgeons. In the future, biomechanical studies must involve more realistic *in vivo *loading conditions. We envisage an approach that involves both experimental and computational methods (see Figure 6). Continuous advancements in the development of ways to measure *in vivo *kinematics of the knee during daily activities are being made. Recently, a dual orthogonal fluoroscopic system has been used to measure *in vivo *knee kinematics, with an accuracy of 0.1 mm and 0.1° for objects with known shapes, positions and orientations [76]. Once collected, the *in vivo *kinematic data can be replayed on cadaveric specimens using the robotics/UFS testing system in order to determine the *in situ *forces in the ACL and ACL grafts. In parallel, subject-specific computational models of the knee can be constructed. Based on the same *in vivo *kinematic data, the *in situ *forces in the ACL and ACL grafts can be calculated. When the calculated *in situ *forces are matched by those obtained experimentally, the computational model is then validated and can be used to compute the stress and strain distributions in the ACL and ACL grafts, as well as to predict *in situ *forces in the ACL and ACL grafts during more complex *in vivo *motions that could not be done in laboratory experiments. In the end, it will be possible to develop a large database on the functions of ACL and ACL grafts that are based on subject-specific data (such as age, gender, and geometry), to elucidate specific mechanisms of ACL injury, to customize patient specific surgical management (including surgical pre-planning), as well as to design appropriate rehabilitation protocols. We believe such a biomechanics based approach will provide clinicians with valuable scientific information to perform suitable ACL reconstruction and design appropriate post-operative rehabilitation protocols. In the end, all these advancements will contribute to better patient outcome.
